# Body composition in adults with phenylketonuria: A 5 years follow-up study

**DOI:** 10.1016/j.ymgmr.2026.101321

**Published:** 2026-05-21

**Authors:** Bianca Fasolo Franceschetto, Yannick Moutapam-Ngamby-Adriaansen, Nathalie Tressel, Arnaud de Luca, Manuel Schiff, Soraia Poloni, Ida Vanessa D. Schwartz, François Maillot

**Affiliations:** a Federal University of Rio Grande do Sul - Postgraduate Program in Medicine: Medical Sciences, Porto Alegre, RS, Brazil; b Department of Internal Medicine, University Hospital of Tours, Tours, France; c University of Tours, INSERM 1253 “iBraiN”, Tours, France; d Department of Nutrition, University Hospital of Tours, Tours, France; e Reference Center for Inborn Errors of Metabolism, Necker University Hospital, APHP and University of Paris Cité, Filière G2M, MetabERN, INSERM UMRS 1163, Institut Imagine Paris, France; f Department of Nutrition, Porto Alegre Clinical Hospital, Porto Alegre, RS, Brazil; g Department of Genetics, Federal University of Rio Grande do Sul, Porto Alegre, RS, Brazil; h InRaras, National Institute of Science and Technology in Rare Diseases, Porto Alegre, RS, Brazil

**Keywords:** Body composition, Phenylketonuria, Phenyalanine-restricted diet, adults

## Abstract

This longitudinal study evaluated body composition in 24 early-treated adults with PKU using Dual-Energy X-ray Absorptiometry (DXA). At baseline, eutrophy, overweight, and obesity were found in 9, 9, and 6 patients, respectively. After 5 years, sixteen patients presented reduction in lean mass and an increase in body fat percentage, despite stable weight and BMI. These adverse changes may be linked to the phenylalanine restricted diet. These findings highlight the need for monitoring and targeted interventions to mitigate body composition deterioration in this population.

## 1 Introduction

Phenylketonuria (PKU) is an inborn error of metabolism caused by biallelic pathogenic variants in the *PAH* gene, leading to deficiency of phenylalanine hydroxylase and resulting in neurotoxic accumulation of phenylalanine. Treatment is based on a natural protein restricted diet supplemented with phenylalanine-free protein substitutes containing all other essential amino acids [1]. Given the restrictive nature of this diet in terms of natural protein, along with an increased intake of carbohydrates and the chronic exposure to synthetic amino acids, monitoring body composition in these individuals is relevant, especially on the long term [2]. Some studies have investigated body composition in PKU, mostly on pediatric populations. Among those that evaluated adults, most are cross-sectional comparing early treated PKU patients to healthy controls [3], [4], [5], [6], and generally reporting no significant differences between groups. Thus, the main objective of this study was to evaluate body composition by Dual-Energy X-ray Absorptiometry (DXA) in adults with PKU at baseline and after five years. Additionally, we assessed the relation between body composition, phenylalanine (Phe) blood levels and protein intake.

## 2 Methods

### 2.1 Study design, inclusion criteria and ethical considerations

This study evaluated the data from a two-center sample of the ECOPHEN study (ClinicalTrials.gov Identifier: NCT01619722) a French national prospective cohort study including early treated (defined as diagnosis through newborn screening programs, with dietary intervention initiated within the first weeks of life) adults (≥18 years) with PKU who provided written informed consent. The University Hospital of Tours and the Robert Debré University Hospital in Paris were the only ones to include DXA evaluation in the ECOPHEN protocol. Exclusion criteria were neurological comorbidities, cancer diagnosis, and late PKU diagnosis. The study was funded by the French national hospital program for clinical research and followed ethical standards of the French committee on human experimentation and the Helsinki Declaration of 1975, revised in 2013.

### 2.2 Data collection

The study included 24 participants who had DXA performed at year 0. Of these, sixteen participants completed both baseline (Y0) and five-year follow-up (Y5) body composition assessments, forming the longitudinal cohort. Data collected included age, sex, body mass index (BMI), weight, height, body fat percentage, fat mass, lean mass, blood Phe levels, and protein intake: natural protein (from food sources only) and total protein intake (from food sources plus phenylalanine-free protein substitutes). Body composition was assessed using Hologic DISCOVERY (Marlborough, Massachusetts, USA) and then HOLOGIC Horizon W DXA. Patients were classified according to their BMI, calculated as weight in kilograms divided by height in meters squared (kg/m^2^). The following categories were considered: normal weight (BMI < 25.0 kg/m^2^), overweight (BMI 25.0–29.9 kg/m^2^), and obesity (BMI ≥ 30.0 kg/m^2^).

### 2.3 Statistical analysis

Quantitative variables are presented as median and interquartile range, categorical variables as absolute and relative frequencies. For cross-sectional analysis, group comparisons were conducted using Mann-Whitney *U* test. For longitudinal analysis, changes over time were evaluated using Wilcoxon signed-rank test. Correlations between variables were examined using Spearman's correlation, cross-sectional data was available at baseline for 24 patients; longitudinal data was available for 16 patients. The statistical significance level was set at α = 0.05 for all analyses. Analyses were performed using JASP (Version 0.95.4).

## 3 Results

**Fig. 1:**
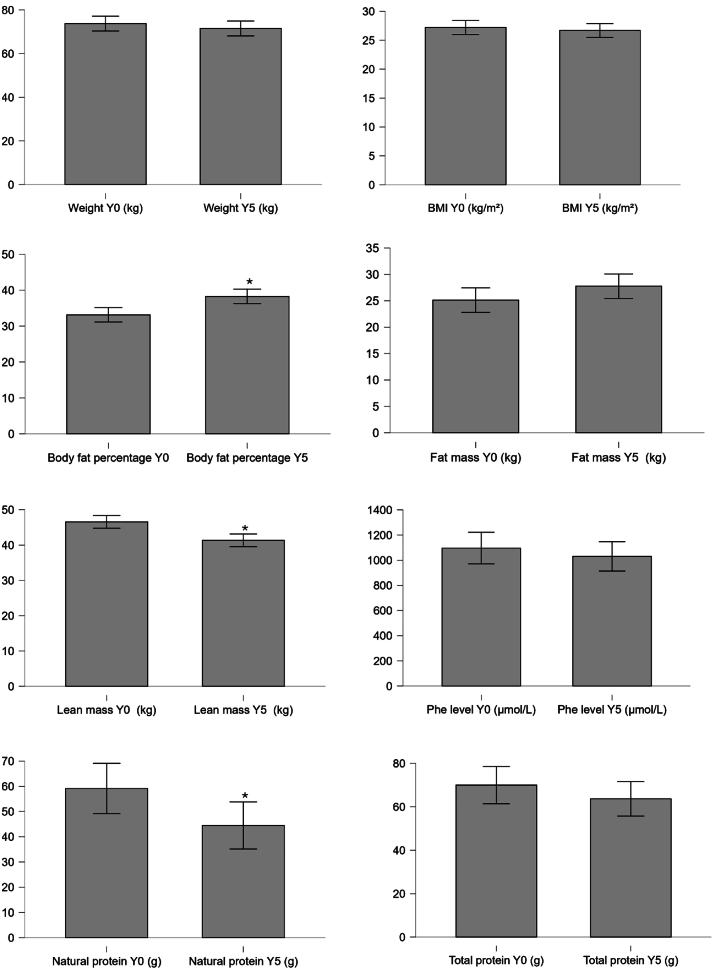
Anthropometric, body composition, phenylalanine levels, and protein intake parameters at baseline (Y0) and after five years of follow-up (Y5) in adults with PKU. Bars represent median values with interquartile range (IQR). Statistical comparisons were performed using the Wilcoxon signed-rank test. * *p* < 0.05 vs. Y0. *n* = 16.

At baseline, we included 18 female patients and 6 males. Nine patients had normal BMI, 9 were overweight and 6 were obese. The comparison between baseline and Y5 (*n* = 16, female = 13, normal BMI = 7, overweight = 6, obesity = 3) revealed no significant weight changes over time (*p* = **0.410**), but a significant decrease in lean mass (*p* = **0.004**), along with a significant increase in body fat percentage (*p* = **0.005**). The lean mass decreased from media of 41.48 kg in Y0 to 40.06 kg in Y5, representing a loss of approximately 0.28 kg/year (Table 1 and Fig. 1). Over the five years, the fat mass did not increase significantly (*p* = **0.051**).

**Table 1:** Baseline demographics and clinical data of the total sample (*N* = 24) and longitudinal subgroup (*N* = 16), with five-year follow-up comparison.

Variable	Median (IQR) N = 24	Median (IQR) Y0 N = 16	Median (IQR) Y5 N = 16	*p* value (Comparison between Y0 and Y5)
Age (years)	30.50 (8.0)	32 (8.5)	37.00 (8.5)	
Weight (kg)	70.50 (23.32)	68.90 (26.97)	71.80 (12.35)	0.410
Height (cm)	167.00 (11.25)	161.75 (8.5)	161.75 (7.75)	0.085
BMI (kg/m^2^)	26.50 (5.39)	26.50 (8.61)	25.66 (5.15)	0.551
Body fat percentage (%)	36.36 (9.45)	36.36 (9.55)	40.55 (12.15)	**0.005**
Fat mass (kg)	25.88 (13.47)	24.78 (14.36)	27.44 (10.62)	0.051
Lean mass (kg)	43.79 (13)	41.48 (13.2)	40.06 (10.28)	**0.002**
Blood Phe level (μmol/L)	1219.00 (684.50)	1219.00 (878.50)	1147.50 (905)	0.421
Natural protein intake (g)	61.05 (29.11)	66.96 (40.19)	42.04 (49.53)	**0.033**
Total protein intake (g)	64.65 (20.51)	71.66 (31.12)	61.36 (14.01)	0.305

Y0 = year 0; Y5 = year 5; BMI = body mass index; Phe = phenylalanine.

The longitudinal design (*n* = 16) also revealed a significant reduction in natural protein intake from baseline to Y5 (0.97 g/kg at Y0 vs. 0.59 g/kg at Y5; *p* = **0.033**). Protein substitute intake remained stable over the same period (*p* = **0.183**). Overall, total protein intake remained unchanged (Fig. 1). Correlation analyses performed in the longitudinal sample, with the differences from baseline to Y5, showed no significant association between body composition changes (decrease in lean mass and increased body fat percentage) and total protein intake (rho = 0.184, *p* = 0.547; rho = 0.403, *p* = 0.172) or natural protein intake (rho = 0.116, *p* = 0.707; rho = 0.442, *p* = 0.130). Also, no correlation with blood Phe levels differences (rho = 0.086, *p* = 0.761; rho = 0.258, *p* = 0.353) were observed. There was no correlation between lean mass loss and total protein intake (rho = −0.519, *p* = **0.057**).

Regarding therapy compliance, blood Phe data was available for 23/24 participants. Blood Phe levels showed wide individual variability (157–2328 μmol/L), with only 5/23 patients (21.7%) within the recommended target of <600 μmol/L. In the longitudinal subgroup (*n* = 16), blood Phe data were available for 15/16 participants at Y0 and 16/16 at Y5. The proportion of patients within targets were 4/15 (26.7%) at Y0 and 5/16 (31.3%) at Y5, with all patients controlled at Y0 remaining within target range at Y5. No significant differences in body composition were observed between patients within and outside the Phe target range at either timepoint (*p* > 0.05 for all comparisons).

Correlations between blood Phe levels and weight (rho = 0.199, *p* = 0.224), and BMI (rho = 0.100, *p* = 0.546), and body fat percentage (rho = −0.183, *p* = 0.264), and fat mass (rho = 0.038, *p* = 0.820), and lean mass (rho = 0.209, *p* = 0.202) were not significant (*n* = 40, 24 and 16 from the cross sectional and longitudinal studies, respectively).

## 4 Discussion

This is the first study to assess long-term body composition changes in adults with PKU. Over five years, we observed a significant decrease in lean mass without weight changes and, as result, an increase in body fat percentage. The median blood Phe levels in our cohort were above the recommended maximum target of <600 μmol/L throughout the study period. However, this reflects the real-life metabolic control commonly observed in adult PKU populations across Europe [7], [8].

In the general population, overweight and obesity risks increase between 20 and 40 years [9], [10]. A French cohort demonstrated that lean mass begins to decrease at 31 years in females and 55 years in males (significant decrease of 0.03 kg/year and 0.16 kg/year respectively) while fat mass progressively increases with age in both sexes [11]. Thus, the recent literature suggests that significant body composition alterations can occur early in adulthood. Our data suggests that these changes in body composition can occur at similar ages but may progress at a substantially faster rate in patients with PKU, as we observed a loss of approximately 0.28 kg/year. Notably, despite stable BMI, we observed shifts in BMI classification, with more participants moving into the normal range, which should be interpreted cautiously, as this coincided with lean mass loss.

Cross-sectional studies comparing PKU patients to healthy controls generally report no significant body composition differences [3], [4], [5], although one study reported higher body fat percentage in female PKU patients [6]. Alongside these findings, the literature notes that, given the rising prevalence of overweight in the general population, longitudinal monitoring of body composition in PKU is essential [2], [4], particularly to prevent adiposity and reduce cardiometabolic risk [12]. It is important to emphasize that body composition changes during lifetime are closely tied to health status. Increase in fat mass and decrease in lean mass have been associated with a rise in age-related pathologies and functional decline [13].

Higher natural protein intake has been associated with greater lean mass and lower fat mass in the adult PKU population [14], though further studies are needed to investigate its long-term effects of protein intake on body composition [12]. In our sample, we observed a significant decrease in natural protein intake at Y5. Concurrently, protein substitute intake increased, from 0.07 g/kg at Y0 to 0.27 g/kg at Y5, reflecting a compensatory effort in the expected clinical direction. However, this compensatory increase was insufficient to meet the revised European PKU guideline minimum of 1 g/kg of total protein (WHO safe level plus 20% additional from protein substitute [15]) at Y5 body weight, approximately 29.5 g/day of protein substitute would have been required, compared to the 19.3 g/day consumed. As a result, total protein intake declined from 1.04 g/kg at Y0 (minimum recommended) to 0.85 g/kg at Y5, falling below the recommended threshold. The moderate negative correlation between total protein intake and lean mass loss (rho = −0.519, *p* = 0.057), while not reaching statistical significance, is consistent with this nutritional mechanism hypothesis. Similarly, blood Phe levels did not correlate significantly with changes in body composition, a finding consistent with other authors [6], [16].

This study has some limitations, including the absence of a healthy control group and the small longitudinal sample size (*n* = 16), which reduces statistical power. However, these are inherent challenges in research on rare metabolic diseases. The longitudinal design with repeated DXA measurements represents a methodological strength that supports the validity of our findings.

## 5 Conclusion

In conclusion, this study provides the first longitudinal evidence of body composition changes in adults with PKU, highlighting a risk profile for reduced lean mass and increased body fat percentage early in adults. Total and natural protein intake appears to play a crucial role that needs to be investigated. Further studies with larger samples are needed to better understand the determinants of these changes and to support the development of targeted interventions, such as adequate nutritional monitoring associated with physical exercise, aimed at mitigating adverse body composition outcomes in this population.

## CRediT authorship contribution statement

**Bianca Fasolo Franceschetto:** Writing – original draft, Methodology, Formal analysis, Data curation. **Yannick Moutapam-Ngamby-Adriaansen:** Writing – review & editing, Methodology. **Nathalie Tressel:** Writing – review & editing, Data curation. **Arnaud de Luca:** Writing – review & editing, Data curation. **Manuel Schiff:** Writing – review & editing, Data curation. **Soraia Poloni:** Writing – review & editing. **Ida Vanessa D. Schwartz:** Writing – review & editing, Methodology. **François Maillot:** Writing – review & editing, Supervision, Investigation, Data curation.

## Declaration of competing interest

None.

## Data Availability

Data will be made available on request.
